# A Large-Scale Assessment of Nucleic Acids Binding Site Prediction Programs

**DOI:** 10.1371/journal.pcbi.1004639

**Published:** 2015-12-17

**Authors:** Zhichao Miao, Eric Westhof

**Affiliations:** Architecture et Réactivité de l'ARN, Université de Strasbourg, Institut de Biologie Moléculaire et Cellulaire du CNRS, Strasbourg, France; University of Missouri, UNITED STATES

## Abstract

Computational prediction of nucleic acid binding sites in proteins are necessary to disentangle functional mechanisms in most biological processes and to explore the binding mechanisms. Several strategies have been proposed, but the state-of-the-art approaches display a great diversity in i) the definition of nucleic acid binding sites; ii) the training and test datasets; iii) the algorithmic methods for the prediction strategies; iv) the performance measures and v) the distribution and availability of the prediction programs. Here we report a large-scale assessment of 19 web servers and 3 stand-alone programs on 41 datasets including more than 5000 proteins derived from 3D structures of protein-nucleic acid complexes. Well-defined binary assessment criteria (specificity, sensitivity, precision, accuracy…) are applied. We found that i) the tools have been greatly improved over the years; ii) some of the approaches suffer from theoretical defects and there is still room for sorting out the essential mechanisms of binding; iii) RNA binding and DNA binding appear to follow similar driving forces and iv) dataset bias may exist in some methods.

## Author Summary

Nucleic acid binding sites in proteins are functionally important in a majority of biological processes. Computationally predicting these binding sites can help the biological community in understanding the nucleic acid binding proteins in the very first step. The emergence of nucleic acid binding site prediction programs and web servers during the last decade shows a great diversity in various aspects. Besides, some binding site prediction related questions, such as i) can RNA binding sites be distinguished from DNA binding sites? ii) can RNA and DNA binding sites be predicted in the same model?, have not been fully answered. Here, we benchmarked 19 web servers and 3 stand-alone programs on 41 previously reported data sets and analyzed the prediction results in different aspects to show a more complete view of how well these programs can perform and how they can be used. We hope to demonstrate some key points for unbiased comparison for further development of similar prediction programs.

## Introduction

Protein-nucleic acid (RNA/DNA) bindings play crucial roles in most biological processes[1] and the detection of the functional sites/regions in proteins is an important step for structurally understanding the molecular mechanism of the biological processes. Compared with the vast number of protein-nucleic acid interactions in bio-systems (**Supplementary Note 1 in S1 Text**), the experimental determination of binding sites is always difficult, demanding and not always readily feasible. Hence, computational prediction of nucleic acid binding sites has been an established field in computational and molecular biology over the past two decades.

The prediction approaches are diverse in many aspects, which results in controversies over technical details and renders difficult to make totally fair comparisons[2]. Further, previous reviews[3–6] only assessed at small scale the available datasets. Currently, RNA- and DNA-binding residue predictions are always treated as different problems, or trained with different data sets within the same model[7–11]. However, whether RNA- and DNA-binding proteins (**Supplementary Note 2 in S1 Text**) exploit different driving forces is not established and it is known that some proteins do bind both types of nucleic acids. Very recently, Yan and coworkers noticed also that prediction programs are unable to distinguish between DNA and RNA binding proteins and concluded that one should compare RNA- and DNA-binding site predictors together[6]. The definition of a nucleic acid binding residue is not standardized with definitions ranging from distance cutoffs[8,9,12–15] to the enumeration of non-covalent contacts[16–19](**Supplementary Note 3 in S1 Text**). This leads to ambiguities goal in the problem and variations in prediction accuracy. Besides, tens of training and test sets of variable sizes have now been curated by developers during the development of computational approaches. How to avoid bias in a dataset is nontrivial. Further, the assessment criteria are still arguable, e.g. whether all residues from different proteins should be compared together (**Supplementary Note 4 in S1 Text**). In addition, programs differ in their approaches (**Supplementary Note 5 in S1 Text**) making a fair assessment difficult. Finally, the distribution and ease-of-use of the programs greatly determine their help to the users in the biological community.

In this report, we present a large-scale assessment of 19 currently available web servers and 3 stand-alone prediction programs in nucleic acid binding site prediction, which is 24 predictors in total, on 41 different datasets derived from structures of protein-nucleic acid complexes in the PDB, including more than 5000 proteins. We use a hierarchical definition of binding sites and various assessment criteria for reference. We analyze differences i) between RNA binding site prediction and DNA binding sites predictions; ii) between binary prediction and continuous scores; iii) between sequence-based prediction and structure-based ones; and finally iv) between original and updated programs. The large-scale analysis should be helpful to developers and users.

## Results

**Fig 1:**
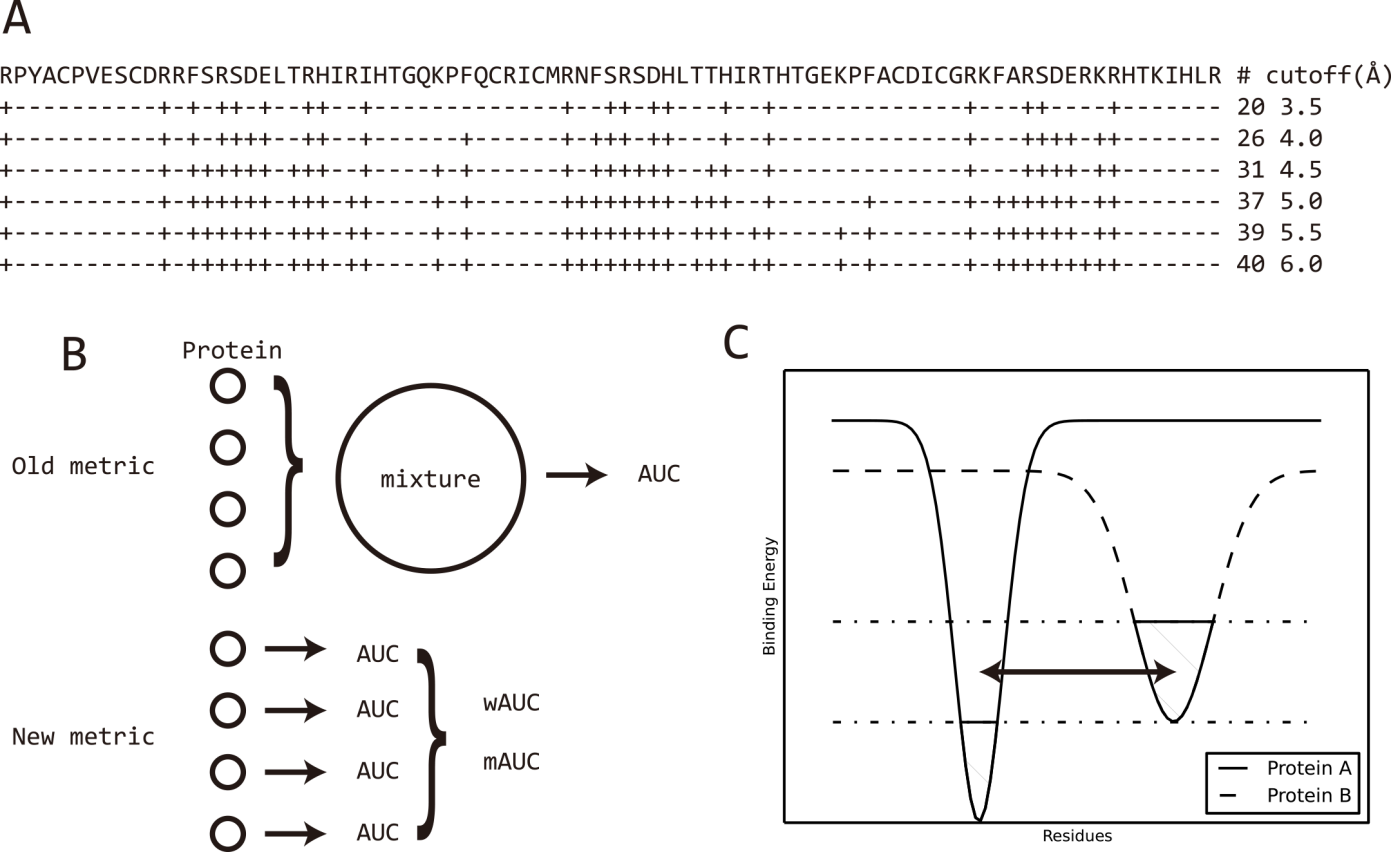
Binding site definition and assessment metrics can result in accuracy variation. A) Binding site definition of Zif268 protein based on different distance cutoffs. ‘+’ marks the binding sites while ‘-’ marks non-binding sites. With a distance cutoff of 6.0Å, 40 residues are defined as binding sites, which is twice that obtained with a cutoff of 3.5Å; B) Two metrics to measure prediction accuracy in terms of AUC. Old metric mix all the residues from all the proteins together for comparison, then measure AUC on the mixed data. Metric in this work measures AUC for each protein and average the AUC values considering protein length. C) A scheme to illustrate the irrelevant comparison between binding sites of a protein and the non-binding sites on another protein. As protein A and protein B may have different size of nucleic acid binding region and binding affinity they are possible to have different energy funnels. The dashed region shows the binding region of the two proteins. Binary assessment, which mixes all residues together, will certainly include comparison between non-binding sites of protein A and binding sites of protein B, shown by the double arrowed line.

The prediction of nucleic acid binding sites is usually determined by three main factors: the definition of a binding site, the assessment criteria and the datasets. Currently, there is no universal definition of a binding site and a minimum distance cutoff between interacting residues is most frequently applied. However, different distance cutoffs lead to accuracy variations while a single cutoff biases certain prediction programs. In **Fig 1A** is displayed a real-world case where a distance cutoff of 6.0Å leads to a two times higher number of binding sites than that obtained with a cutoff of 3.5Å. **S1 Table** and **S12 Fig** show that this difference is a general distribution rather than a rare case. And, in **S2 Table**, the data show that with a cutoff of 3.5Å used for prediction and 6Å for the definition of the binding sites, the final specificity is 100% but the sensitivity is as low as 51–62% (high false negative rate) with a total accuracy (ACC) decrease down to ~90% (details are discussed in **Supplementary Note 6 in S1 Text**). To fully capture the accuracy variance resulting from a distance cutoff, a hierarchical definition with distance cutoffs ranging from 3.5 to 6Å using 0.5Å as step was used and the distributions of the accuracies were plotted. Besides, the prediction accuracy highly depends on the dataset used for testing. Normally, the largest the training set the better the prediction model, thus making it difficult to compare different programs. However, a good prediction program should show stable accuracy on all the datasets, a feature that cannot be achieved by biased predictions even with a large training set (**Supplementary Note 7 in S1 Text**). We used 41 datasets to accentuate the possibility of biased prediction by the programs. Finally, different criteria are measured to show different aspects of the programs. The webservers assessed include BindN[8], BindN+[9], RNABindR[20], RNABindRPlus[21], DBS-Pred[12], DBS-PSSM[13], KYG[14], PRBR[22], PPRInt[23], DNABINDPROT[24], ProteDNA[25], DISPLAR[10], DR_bind1[26], aaRNA[27], RBscore[28], RBRDetector[29], DNABind[30], xypan[31] and RNAProSite (lilab.ecust.edu.cn/NABind/), while the programs are Predict_RBP[17], PRNA[32] and RBRIdent[33]. Previously reported prediction approaches have been summarized in **Table 1**. Slow programs DR_bind1 and RBRDetector were only tested on part of the datasets and the results are provided in the supplementary information. metaDBSite[34] shows same result as BindN and was not tested explicitly.

**Table 1:** Summary of the existing approaches in nucleic acid binding site prediction.

RNA																	
Sequence-based						Brief/Features											
Year	Name	Server	Program	Website	Binding Site Definition	PSSM	RP	ASA	HP	SS	EC	Q	SA	Training	window	Dataset	reference
2006	BindN	✓		http://bioinfo.ggc.org/bindn/	3.5Å				✓			✓		SVM	11	R107(PRINR25),D62(PDNA-62)	[8]
2007	RNABindR	✓		http://einstein.cs.iastate.edu/RNABindR/	5Å	✓								NB	25	R147	[20]
2008	PPRInt	✓		http://www.imtech.res.in/raghava/pprint/	6Å	✓	✓							SVM	17	R107 from BindN, R86 from PPRInt	[23]
2008	RNAproB				6Å/3.5Å	✓								SVM	25	R107 from BindN, R86 from PPRInt, R109 from RNABindR	[35]
2008	PRINTR	✓		http://210.42.106.80/printr/	ENTANGLE	✓								SVM	15	R109 from RNABindR	[16]
2008	RISP	✓		http://grc.seu.edu.cn/RISP	3.5Å	✓								SVM	7	R147 from RNABindR, R71(PRNA-71,NA)	[36]
2009	PiRaNhA	✓		http://www.bioinformatics.sussex.ac.uk/PIRANHA	3.9A	✓	✓	✓	✓					SVM	23	R81 from PPRint,R42(NA)	[37]
2010	BindN+	✓		http://bioinfo.ggc.org/bindn+/	3.5Å	✓			✓			✓		SVM	11	R107(PRINR25),D62(PDNA-62)	[9]
2010	NAPS	✓		http://proteomics.bioengr.uic.edu/NAPS/	4.5Å	✓	✓							DT	7	R109 from RNABindR, D84 from Pro-dna, D274 from DISIS, D62(PDNA-62)	[7]
2010	PRBR	✓		http://www.cbi.seu.edu.cn/PRBR/optional.php	3.5Å	✓	✓							RF	11	R180 (RBP-180)	[22]
2011	Sungwook				H-bond			✓	✓			✓		SVM	9	R3149(PRI3149),R727(PRI727),R267(PRI267)	[38]
2011	SRCPred	✓		http://tardis.nibio.go.jp/netasa/srcpred/	3.5Å	✓								NN	5	R160(PRNA160)	[39]
2011	Predict_RBP		✓	cic.scu.edu.cn/bioinformatics/Predict_RBP.rar	ENTANGLE	✓	✓	✓						SVM	15	R107 from BindN, R86 from PPRInt, R109 from RNABindR	[17]
2011	meta2	✓		https://genesilico.pl/meta2	3.5Å	Meta-server										R44, R38 from OPRA, R180 from PRBR, R111 from RNABindR, R81 from PiRaNha, R86 from KYG	[4]
2012	Qian-Zhong				6Å/3.5Å/ENTANGLE	✓		✓						SVM	25	R107 from BindN, R86 from PPRInt, R109 from RNABindR	[40].
2014	RNABindRPlus	✓		http://einstein.cs.iastate.edu/RNABindRPlus/	5Å	✓								SVM	21	R28,R44,R111,R198	[21]
2015	RBRIdent		✓	http://166.111.152.91/RBRIdent	ENTANGLE	✓				✓		✓		RF	9	R281	[41]
**Structure-based**																	
2006	KYG	✓		http://cib.cf.ocha.ac.jp/KYG/	7Å	✓	✓							Function		R86	[14]
2008	RsiteDB	✓		http://bioinfo3d.cs.tau.ac.il/RsiteDB/	7Å								✓	Clustering			[15]
2008	DR_bind1	✓		http://drbind.limlab.ibms.sinica.edutw/	HBPLUS			✓			✓	✓		Function		D56 from Susan 2003, D69, R81	[42]
2009	PRIP	✓		http://www.qfab.org/PRIP	5Å	✓		✓						SVM	19	R147(R144) and R109 from RNABindR	[43]
2010	OPRA		By contact		4Å	statistical potentials								Function		R316,R38	[44]
2011	DRNA	✓			4.5Å								✓	Function		R250(RB250),R212(RB212),RBD292(NA)	[45]
2010	Struct-NB				5Å		✓							NB		R147 from RNABindR	[46]
2010	PRNA		✓	http://doc.aporc.org/wiki/PRNA	ENTANGLE	✓	✓	✓	✓					RF	5	R205	[32]
2014	aaRNA	✓		http://sysimm.ifrec.osaka-u.ac.jp/aarna/	3.5Å	✓		✓		✓	✓			NN	11	R67,R141,R205	[27]
2014	RBRDetector	✓		http://ibi.hzau.edu.cn/rbrdetector	4.5Å, 10%rASA	✓								SVM	11	R264, R75	[29]
2014	Xiaoyong				ENTANGLE	✓		✓		✓		✓		RF	5	R205(PRNA)	[31]
2015	RBscore	✓		http://ahsoka.u-strasbg.fr/rbscore/	3.5-6Å		✓	✓			✓	✓				R130,R116	[28]
2015	RNAProSite	✓		lilab.ecust.edu.cn/NABind/													
**DNA**																	
**Sequence-based**																	
2004	DBS-Pred	✓		http://www.abren.net/dbs-pred/	3.5Å		✓	✓		✓				NN	3	D62(PDNA-62), NRTF-915	[12]
2005	DBS-PSSM	✓		http://www.abren.net/dbs-pssm/	3.5Å	✓								NN	5	D62(PDNA-62), PDNA-RDN(NA), PDNA-NR90(NA)	[13]
2006	DNABindR				ΔASA>1			✓	✓	✓	✓	✓		NB	9	D171	[47]
2007	DISIS	✓		http://cubic.bioc.columbia.edu/services/disis	6Å	✓		✓		✓				SVM	9	D274	[48]
2007	DP-Bind	✓		http://lcg.rit.albany.edu/dp-bind/	3.5Å	✓								kernel regression		D62(PDNA-62)	[49]
2009	ProteDNA	✓		http://protedna.csbb.ntu.edu.tw/	4.5Å	✓								SVM, SSEA	11	D253	[50]
2009	DbindR	✓		http://www.cbi.seu.edu.cn/DBindR/DBindR.htm	3.5Å	✓				✓				RF	11	D374	[51]
2009	SDCPred	✓		http://sdcpred.netasa.org/	3.5Å	✓	✓							NN	5	D159(PDNA159)	[52]
2014	Byungkyu				H-bond		✓							SVM	9	D143	[53]
**Structure-based**																	
1999	Hidetoshi				3.5Å									Function		D52	[54]
2003	Susan				ΔASA>1							✓		Patch analysis		D56	[55]
2005	DBS-kernel				4.5Å		✓					✓		SVM		D83(NA)	[56]
2005	Pro-dna	✓		bioinformatics.bioengr.uic.edu/pro-dna/	4.5Å			✓		✓		✓		SVM		D99 (D96,D50)	[57]
2005	PreDs	✓		http://pre-s.protein.osaka-u.ac.jp/~preds/	3.0Å							✓		Function		D63	[58].
2007	DISPLAR	✓		http://pipe.scs.fsu.edu/displar.html	5Å	✓								NN	15	D428	[10]
2007	DR_bind1	✓		http://drbind.limlab.ibms.sinica.edu.tw/	HBPLUS	Energy based								Function		D56 from Susan 2003, D69, R81	[26]
2008	DBD-Hunter	✓		http://cssb.biology.gatech.edu/skolnick/webservice/DBD-Hunter/	4.5Å								✓	Function		D179(DB179)	[59]
2010	DNABINDPROT	✓		http://www.prc.boun.edu.tr/appserv/prc/dnabindprot/	NUCPLOT		✓				✓			GNM	3	D54	[24]
2011	metaDBSite	✓		http://projects.biotec.tu-dresden.de/metadbsite/	3.5Å	Meta-server								NA		D316(PDNA-316),D232(PDNA-232)	[34]
2011	Xiong				4.5Å, 10%rASA	✓						✓		SVM	11	D206	[60]
2012	Sucharita				ΔASA>0.1						✓			SVM		D130(NA)	[61]
2013	Duo-Duo				4.0Å	✓								SVM	11	D62(PDNA-62)	[62].
2013	PreDNA	✓		http://202.207.14.178/predna/	3.5Å	✓		✓					✓	SVM	11	D62(PDNA-62), D224	[63]
2013	DNABind	✓		http://mleg.cse.sc.edu/DNABind/	4.5Å, 10%rASA	✓		✓						SVM, template	11	D206	[30]
2014	Bi-Qing				6Å	✓	✓			✓				SVM	9	D90	[64]

PSSM: position specific scoring matrix derived from sequence alignment

RP: residue propensity

ASA: accessible surface area

HP: hydrophobicity

SS: secondary structure

EC: conservation entropy

Q: electrostatic/pKa

SA: structural alignment

### Overall prediction performance of accuracy and stability

**Fig 2:**
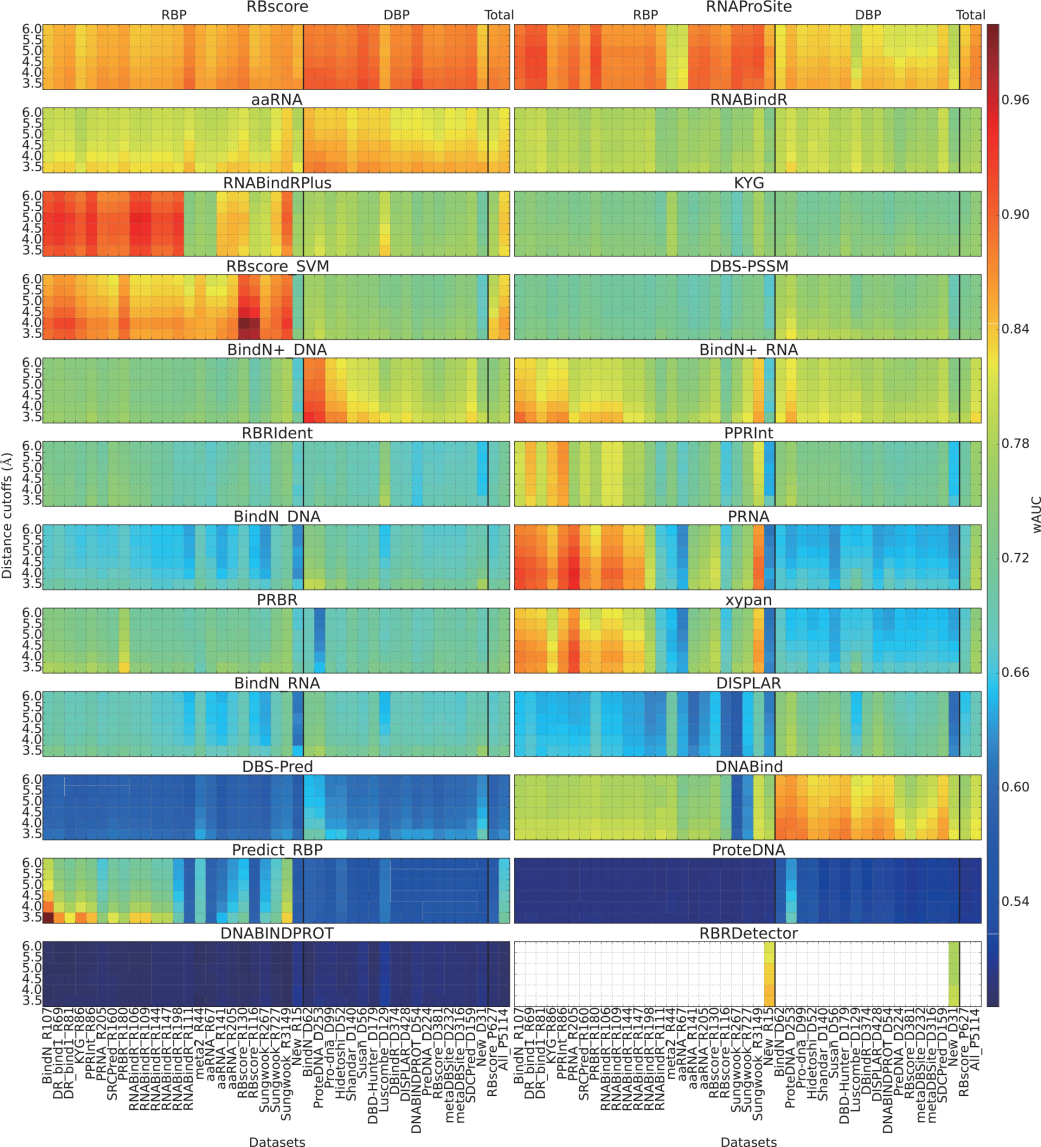
General accuracy distribution based on wAUC. wAUC as assessment criterion of all programs on all datasets with hierarchical definition of binding sites from 3.5 to 6Å. wAUC is the weighted arithmetic mean of AUC and is considered as the criterion to assess the prediction accuracy of the predictors. It is plotted as rainbow colors from highest accuracy of red to lowest accuracy of blue. Each grid in the plot show the wAUC of a predictor (subtitle) on a certain data set (x-axis) assessed according to the binding sites defined by a certain distance cutoff (y-axis). For each subplot, DBP data sets and RBP data sets are separated by bold line. The last two data sets are mixed with DBP and RBP. RBscore_P627 is a non-redundant data set by removing cases of sequence identity >25%. All_P5114 is a mixture of all data sets.

As demonstrated in Fig 1B and 1C, the standard way to measure the area under the receiver operating characteristic (ROC) curve (AUC) would include irrelevant comparisons between binding sites on one protein and non-binding sites on another protein. We assessed the nucleic acid binding site prediction ability by calculating the AUC for each protein and average AUCs on a dataset (wAUC or mAUC, **Supplementary Note 4 in S1 Text**). **Fig 2** demonstrates a general assessment result in terms of wAUC (mean AUC considering the protein length), while mAUC (mean AUC) and tAUC (total AUC that compare all proteins together) are found in **S1 Fig** and **S2 Fig**. Although fluctuations are found, the general distributions of wAUC and mAUC are similar. BindN+, RNABindRPlus, aaRNA, RNAProSite and RBscore rank at the top while DNABind works well on DNA binding protein (DBP). Obviously, dataset bias is a very severe problem for some predictors and require special attention.

**Fig 3:**
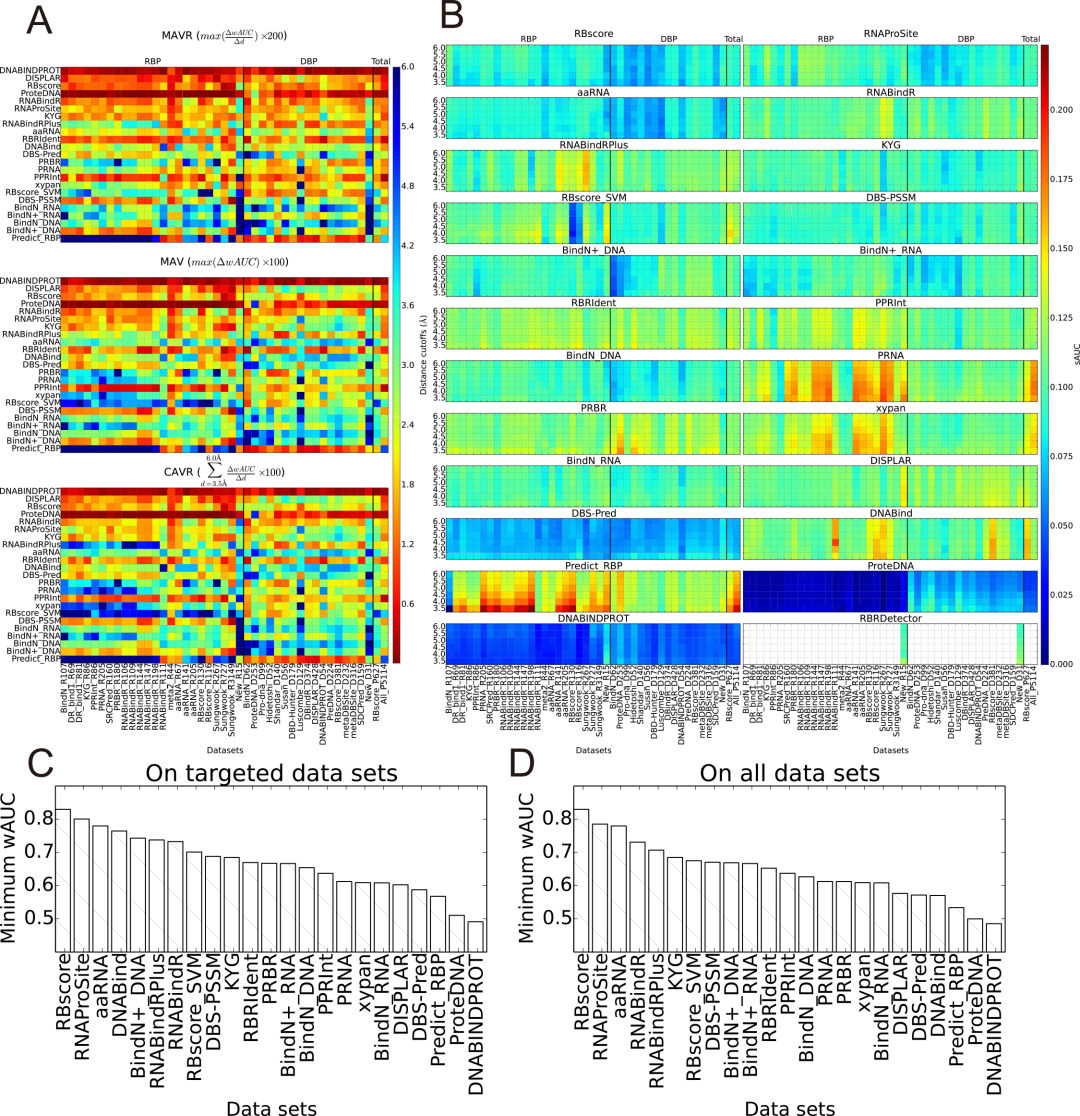
Accuracy variations resulted from binding site definition and data set bias. A) Accuracy variation resulted from distance cutoff based definition of binding sites. Three metrics, MAVR, MAV and CAVR (see methods for details), were used to describe the distance cutoff resulted accuracy variation. The higher the values are, the less stable the predictor is, indicating a less reliable predictor; B) Standard deviation of AUC (sAUC) on all data sets. Similarly, the higher values of sAUC indicate an unstable predictor that cannot guarantee stable accuracy or it is more likely to have data set bias; C) Minimum wAUC of all programs on their targeted data sets: DNA binding site prediction programs are tested on DBP and minimum wAUC are plotted, while the same for RNA binding site prediction programs on RBP. As prediction accuracy of a predictor can vary with data set of assessment and distance cutoff to define binding sites, minimum wAUC demonstrates the bottom line of accuracy that a predictor can guarantee. D) Minimum wAUC of all programs on all data sets.

A successful prediction program should demonstrate stable predictive ability on all the criteria of the assessment. Three criteria (MAVR, MAV and CAVR, described in **Methods**), stand for distance cutoff dependent accuracy variances (wAUC) were used to assess the stability. It can be deduced from **Fig 3A** that the prediction KYG, RNAProSite, RNABindR, ProteDNA, RBscore, DISPLAR and DNABINDPROT are less dependent on distance cutoff (mAUC and tAUC based results could be found in **S3 Fig** and **S4 Fig**). Generally, programs tend to favor the distance cutoff used during training (**Supplementary Note 11 in S1 Text**). In terms of data set, standard deviations of AUC (sAUC) were measured as criteria to assess the stability and is shown in **Fig 3B**. The accuracies of DBS-PSSM, RBscore, aaRNA, DBS-Pred, ProteDNA and DNABINDPROT are more stable when varying the assessment data sets. Considering both prediction accuracy and stability, the ‘barrel effect’ applies to the predictions with dataset bias and a program can best be assessed by its minimum accuracy value in all the datasets. In **Fig 3C**, RNA binding site predictors are assessed on RBP while DNA binding predictors on DBP, the minimum wAUC measured by all distance cutoffs is taken as their prediction ability. **Fig 3D** shows the minimum wAUC applied on all the datasets. A more complete list can be found in **Table 2**.

**Table 2:** Minimum performance of all programs.

DBP datasets												
Sequence-based												
Programs	Distance Cutoff	Dataset	wAUC	mAUC	sAUC	tAUC	SEN	SPC	PPV	ACC	F1	MCC
DBS-Pred	6	RBscore_D381	-	-	-	-	0.418	0.781	0.214	0.736	0.283	0.154
ProteDNA	6	RBscore_D381	-	-	-	-	0.029	0.999	0.799	0.878	0.055	0.137
BindN+_DNA	6	New_D31	**0.743**	0.780	0.091	0.776	0.421	0.908	0.358	0.856	0.387	0.307
RNABindR	6	New_D31	**0.731**	0.753	0.116	0.779	0.648	0.759	0.246	0.747	0.356	0.280
RNABindRPlus	6	New_D31	**0.707**	0.732	0.117	0.759	0.229	0.956	0.390	0.878	0.289	0.236
DBS-PSSM	5.5	New_D31	0.688	0.728	0.111	0.748	0.541	0.825	0.265	0.795	0.356	0.273
RBscore_SVM	6	New_D31	0.684	0.703	0.104	0.737	0.000	1.000	0.000	0.892	0.000	0.000
BindN_DNA	6	Luscombe_D129	0.654	0.678	0.093	0.680	0.341	0.856	0.282	0.783	0.308	0.182
RBRIdent	4.5	New_D31	0.652	0.683	0.104	0.655	0.105	0.953	0.167	0.883	0.129	0.071
PPRInt	4	New_D31	0.652	0.691	0.105	0.690	0.450	0.799	0.148	0.774	0.222	0.155
xypan	6	DNABINDPROT_D54	0.635	0.628	0.109	0.641	0.092	0.982	0.469	0.850	0.153	0.156
PRNA	6	New_D31	0.626	0.653	0.110	0.646	0.336	0.866	0.234	0.809	0.276	0.173
PRBR	6	ProteDNA_D253	0.612	0.587	0.139	0.602	0.317	0.824	0.362	0.702	0.338	0.148
Predict_RBP	4.5	New_D31	**0.534**	0.554	0.130	0.556	0.000	1.000	0.000	0.917	0.000	0.000
Structure-based												
DISPLAR	6	New_D31	-	-	-	-	0.359	0.939	0.416	0.876	0.386	0.318
DNABINDPROT	5.5	BindN_D62	-	-	-	-	0.075	0.929	0.233	0.737	0.113	0.005
RBscore	6	New_D31	**0.837**	0.839	0.089	0.843	0.438	0.918	0.395	0.866	0.415	0.341
aaRNA	6	New_D31	**0.804**	0.813	0.060	0.834	0.552	0.891	0.381	0.854	0.451	0.378
RNAProSite	6	Luscombe_D129	**0.785**	0.803	0.081	0.790	0.760	0.696	0.290	0.705	0.420	0.329
DNABind	6	RBscore_D381	**0.765**	0.798	0.152	0.774	0.614	0.898	0.492	0.859	0.546	0.467
KYG	6	DNABINDPROT_D54	0.707	0.703	0.074	0.712	0.444	0.802	0.281	0.749	0.344	0.206
RBP datasets												
Sequence-based												
DBS-Pred	6	aaRNA_R141	-	-	-	-	0.359	0.790	0.190	0.738	0.248	0.116
ProteDNA	4.5	Sungwook_R267	-	-	-	-	0.001	0.999	0.140	0.888	0.002	0.003
RNABindRPlus	6	RNABindR_R111	**0.738**	0.725	0.113	0.720	0.321	0.914	0.316	0.848	0.319	0.233
RNABindR	6	New_R15	**0.733**	0.756	0.093	0.737	0.661	0.681	0.279	0.678	0.393	0.258
RBscore_SVM	6	New_R15	0.687	0.697	0.135	0.697	0.046	0.987	0.396	0.839	0.083	0.090
DBS-PSSM	5.5	New_R15	0.670	0.695	0.089	0.672	0.473	0.780	0.277	0.734	0.349	0.207
RBRIdent	4	New_R15	0.670	0.699	0.127	0.671	0.169	0.952	0.313	0.863	0.219	0.160
PRBR	6	New_R15	0.667	0.680	0.112	0.664	0.305	0.877	0.317	0.787	0.311	0.185
BindN+_RNA	6	New_R15	0.667	0.687	0.089	0.672	0.378	0.835	0.300	0.763	0.334	0.194
PPRInt	5.5	New_R15	0.637	0.658	0.131	0.648	0.356	0.814	0.255	0.745	0.297	0.150
PRNA	6	New_R15	0.612	0.629	0.141	0.624	0.295	0.865	0.290	0.775	0.293	0.159
xypan	6	New_R15	0.609	0.631	0.106	0.620	0.115	0.981	0.534	0.845	0.189	0.193
BindN_RNA	4	New_R15	0.608	0.642	0.121	0.626	0.324	0.842	0.209	0.783	0.254	0.139
Predict_RBP	6	New_R15	**0.568**	0.581	0.155	0.576	0.022	1.000	1.000	0.846	0.043	0.136
Structure-based												
DISPLAR	6	Sungwook_R267	-	-	-	-	0.214	0.956	0.433	0.856	0.287	0.234
DNABINDPROT	5	Sungwook_R3149	-	-	-	-	0.038	0.948	0.239	0.677	0.066	-0.029
DR_bind1	4.5	meta2_R44	-	-	-	-	0.285	0.942	0.655	0.758	0.397	0.311
RBscore	6	KYG_R86	**0.830**	0.845	0.095	0.862	0.502	0.936	0.697	0.837	0.584	0.496
RNAProSite	6	meta2_R44	**0.801**	0.805	0.101	0.798	0.675	0.770	0.573	0.740	0.620	0.427
aaRNA	5.5	New_R15	**0.780**	0.785	0.070	0.777	0.539	0.854	0.396	0.806	0.457	0.348
KYG	6	Sungwook_R267	0.685	0.690	0.073	0.685	0.362	0.816	0.235	0.755	0.285	0.150
DNABind	4.5	Sungwook_R267	0.570	0.608	0.149	0.600	0.260	0.827	0.169	0.759	0.205	0.073

-: binary predictors and AUC not applicable.

### Detailed comparisons

Some programs tested here are only designed to predict RNA binding sites while others DNA binding sites. However, when tested together, we find that some of the programs show predictive ability on both types of proteins. For instance, RNABindR, KYG, aaRNA, RNAProSite and RBscore are developed on RBP and never trained on DBP, but they also show predictive ability on DBP. In terms of sequence-based methods, RNABindR even demonstrates higher prediction accuracy than most DNA-binding site predictors. aaRNA and RBscore show even higher accuracies for DBP than for RBP. The RNA binding site prediction mode of BindN+ also shows prediction ability on DBP, but its DBP prediction mode has a much lower accuracy on RBP.

**Fig 4:**
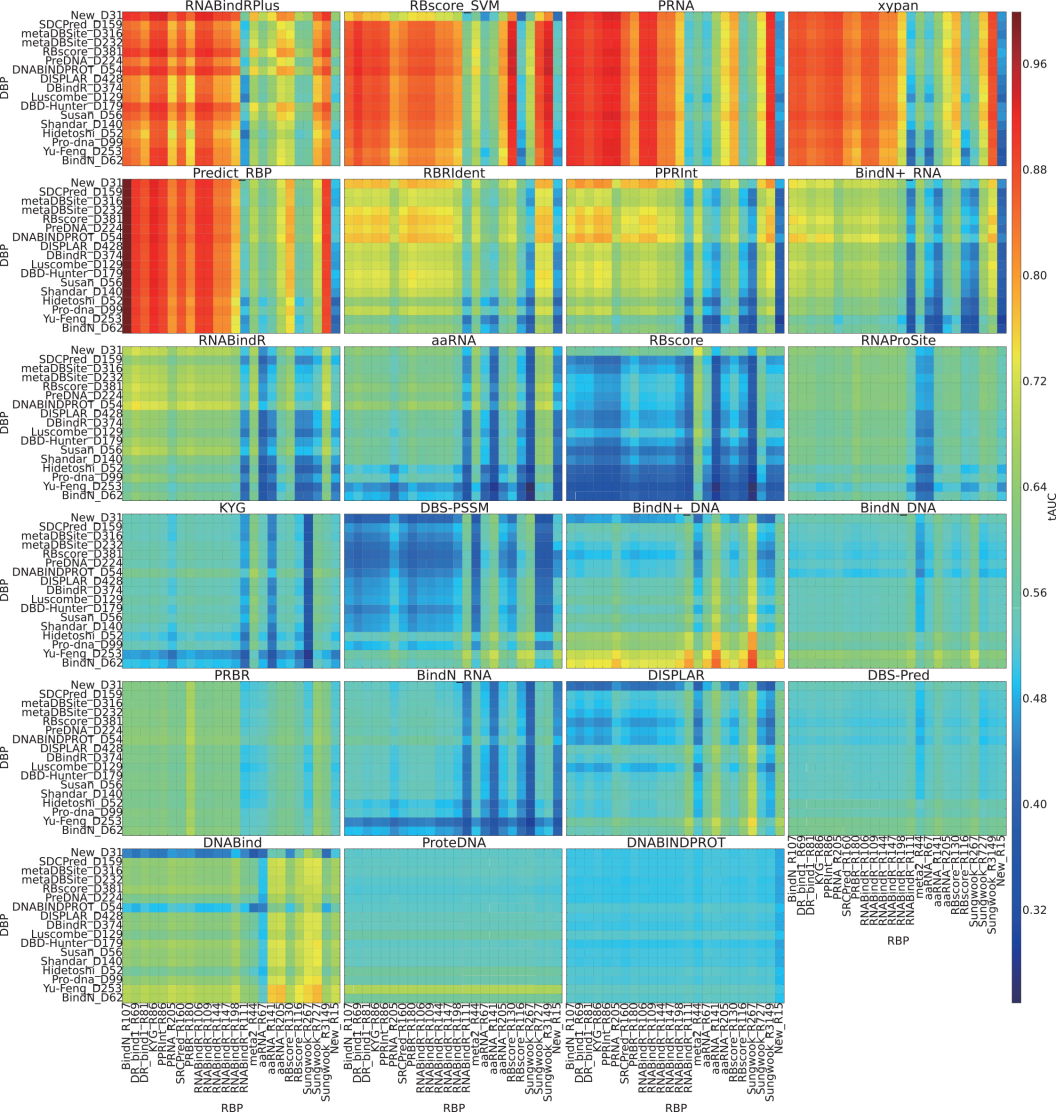
Discrimination between RNA binding sites and DNA binding sites. For DNA binding site prediction programs, DNA binding sites (3.5Å as distance cutoff) from a data set are taken as positive while RNA binding sites from another data set are taken as negative, the AUC values of these discriminations are plotted with different colors. RNA binding site prediction programs take the opposite. Each grid on the heat map show the assessment result in terms of AUC on such a DNA binding sites vs. RNA binding sites data set. i.e. The grid BindN_R107(x-axis)-Susan_D56(y-axis) of program PRNA is to use all the RNA binding sites of BindN_R107 data set as positive while using all DNA binding sites of Susan_D56 data set as negative and measure the AUC value of program PRNA.

Together with the problem of binding site prediction, it is very interesting to find out whether there is a program that can discriminate RNA binding residues from DNA binding residues. This discrimination require three assumptions: i) residues from different proteins can be compared; ii) RNA and DNA binding is driven by different driving forces; iii) such driving forces have been explored by current programs. Previous work from Yan et al.[6] analysed the cross-prediction between RNA- and DNA-binding residue predictors and concluded that they are unable to properly separate DNA- from RNA-binding residues. We performed a more explicit large-scale test and assessed this discrimination by mixing DNA binding residues of a data set with RNA binding residues of another data set. According to **Fig 4**, we find several machine learning based approaches display a discriminative ability for RNA binding residues, including PRNA, Predict_RBP, RNABindRPlus and RBscore_SVM. However, this cannot guarantee predictive ability, since all of the programs have AUC <0.5 on some data sets, which means the programs favor the wrong type of residue. Therefore, we come to the same conclusion than reached by Yan et al. [6], i.e. that none of the current existing predictors can properly distinguish DNA-binding residues from RNA binding ones. Further, we find that the programs PRNA, Predict_RBP, RNABindRPlus and RBscore_SVM have similar distribution for this test while their wAUC distributions on **Fig 2** are also similar. This result implies that these methods have similar prediction accuracies and similar preferences on datasets.

Comparing the updated webservers with the old ones, we find obvious improvement over the years. BindN+ shows consistent improvement over BindN, when programs integrating an homologous search approach, RNABindRPlus, DNABind and RBRDetector show accuracy increase on some of the datasets. New upcoming webservers, aaRNA, RNAProSite and RBscore, show the top ranking performances. Still, xypan and RBRIdent, both improved versions of PRNA, do not display enough effectiveness in this large-scale test.

The AUC is a criterion of assessment that presents a bias towards the score-based predictions rather than binary predictions (binding or not-binding). However, some programs, such as DISPLAR, include a predefined prediction process and give only binary results. On the contrary, in order to obtain binary predictions, some score-based programs only include arbitrary cutoffs, which are not favored by the binary assessment criteria. In a binary comparison, we have to mix up all the proteins in assessment and balance between specificity and sensitivity. Traditional binary assessment criteria are used, including specificity (**S5 Fig**), sensitivity (**S6 Fig**), precision (**S7 Fig**), accuracy (**S8 Fig**), F1 score (**S9 Fig**) and Matthew correlation coefficiency (**S10 Fig**). A general summary of minimum performance is listed in **Table 2**.

Comparing structure-based predictors with sequence-based ones in **Table 2** and **Fig 2**, we can easily find out that some structure-based predictors, aaRNA, RBscore and RNAProSite, are consistently better in performance regardless of the nucleic acid type. This implies that sequence-based approaches that attempt to incorporate predicted structure features such as solvent accessibility and electrostatics cannot capture the real structural features that govern nucleic acid binding. Many sequence-based predictors do not guarantee an AUC above 0.7, which can hardly be considered as meaningful predictions, since an AUC ~0.5 is equivalent random guess.

We find that aaRNA shows similar level of wAUC on dataset meta_R44 (0.82) and Sungwook_R267(0.83), but its sensitivity on meta_R44 (0.8) is much higher than on Sungwook_R267(0.52) while specificity shows the opposite, 0.73 vs. 0.89. Similar cases could also be found in many other programs. In fact, these programs show stable prediction accuracies on both of the sets, but the binary defined sensitivity is a trade-off of specificity and determined by a pre-set cutoff. DNABINDPROT and DISPLAR show top rank specificities and accuracies, which was not described by the AUC distribution. However, when regard to sensitivity, DNABINDPROT ranks at the bottom and DISPLAR is lower than median. This implies that these programs sacrifice sensitivity to gain specificity and accuracy, leading to low true positive rates. This demonstrates that proteins in different environments can have different affinities to nucleic acids and vary in the size of their binding interface. Thus, the use of fixed cutoffs to define binary prediction may misinterpret the binding reality (**Supplementary Note 4 in S1 Text**).

### Can the predictions represent a binding funnel around the binding interface?

**Fig 5:**
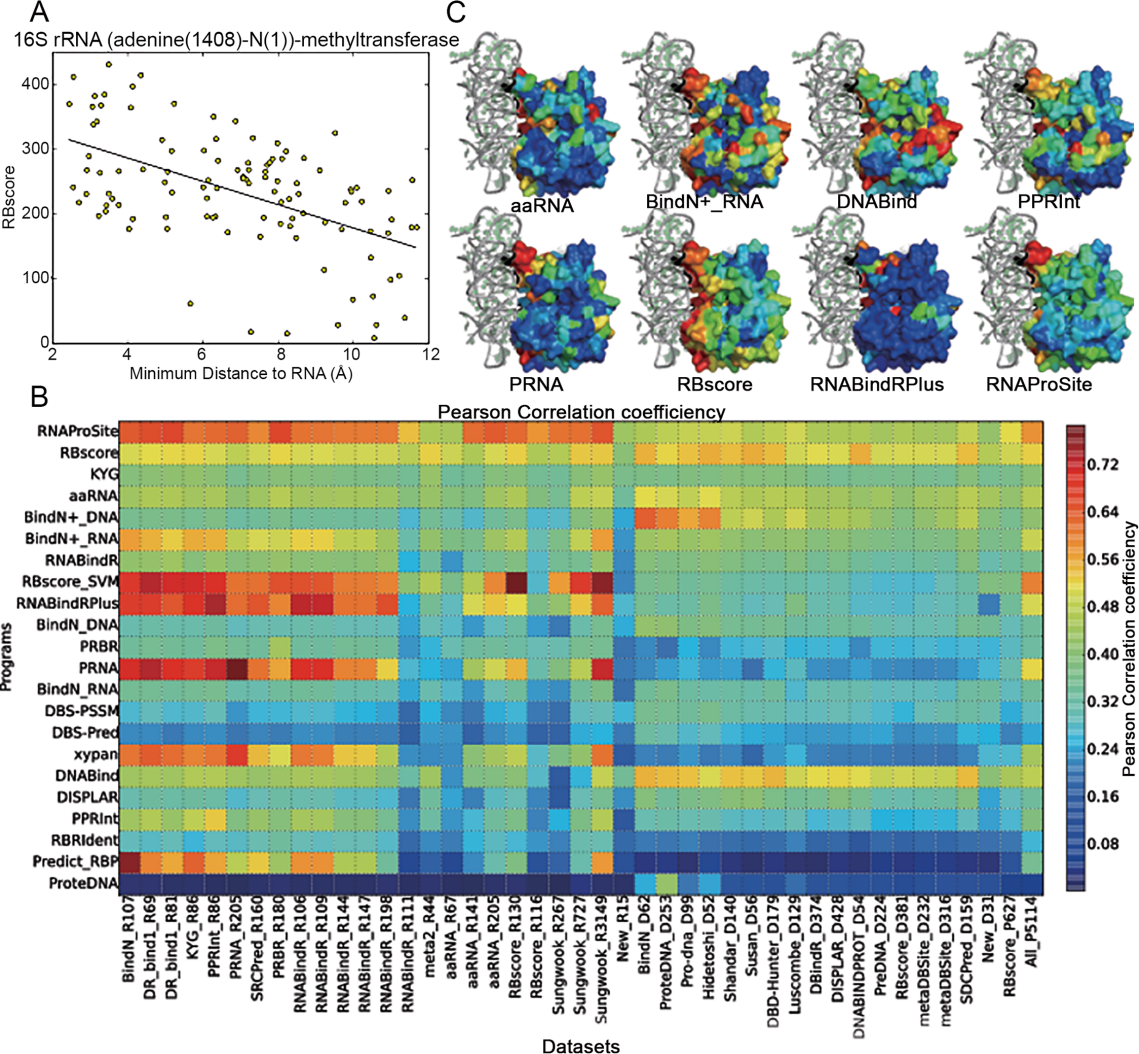
Correlation between prediction score and nucleic acid binding funnel on protein surface. A) Relationship between the minimum distance from a residue around the binding interface (within 12Å) to its RNA ligand (x-axis) and the RBscore of the residue (y-axis). Generally, the RBscore drops when the distance to RNA ligand increases. B) Pearson correlation coefficient, color-coded in rainbow colors, between minimum distance from a residue around the binding interface (within 12Å) to its RNA ligand and the prediction score. The higher Pearson correlation coefficient a predictor has, the more likely its prediction score can display the energy funnel of nucleic acid binding. C) Examples of prediction scores plotted on protein (16S rRNA (adenine(1408)-N(1))-methyltransferase) surfaces as rainbow color, higher binding score region are shown in red.

When we color the protein surface residues with the prediction scores as hierarchical colors, we find that the prediction score of some non-binding residues near the binding region are lower than that of the binding residues but higher than other non-binding ones, **Fig 5A**. The residues around a protein surface are more likely to form a binding funnel than abruptly change from binding region to non-binding ones, which leads to the conclusion that the use of only binary definition and single distance cutoff in the assessment is not feasible. As the residues can gradually change from binding to non-binding region, there could be a correlation between the distance from a residue to the core binding region and the predicted binding score. In **Fig 5B**, the distance to the core binding region is partly represented by the distance to the RNA ligand, and we do find that such a correlation exists in residues around the binding region (within 12Å from the RNA ligands). Although this region maybe smaller for small proteins and the correlation is not necessarily linear, we can roughly measure the correlation by the Pearson correlation coefficient. **Fig 5C** illustrates this distribution for some programs. We find that the programs of higher accuracies also display higher Pearson correlation coefficients. This assessment could be further optimized if the distance to the core binding region is well defined.

### Tests on newly solved structures

Another way to show the predictive ability is to test the newly solve complex structures, since most of the predictors were developed on datasets before 2013. We, thus, collected the protein-nucleic acid complexes solved after 2014, 31 DBP and 15 RBP, as test sets. Because these cases are non-homologous with all the training and test sets of all the programs, they can be taken as an independent test set. This test is similar to the blind tests of CASP[65], *RNA*-Puzzles[66,67] and CAFA[68]. If a prediction program really has predictive ability rather than the ability of interpolation, it should show high accuracy on these new data. The homologous search approaches of some programs, such as RNABindRPlus and DNABind, could be excluded by this assessment. According to **Fig 2**, we find that some of the programs show much lower accuracies on these new data than expected according to previous performance (**Supplementary Note 7 in S1 Text**). But, overall, the results on the newly published data sets are similar to the performance on some ‘difficult sets’ such as meta_R44 and RBscore_R117. The values presented in **Table 2** indicate also that many machine learning based methods show their minimum performance in these two new datasets. This highlights the data set bias problem of the machine learning approaches that can only demonstrate predictive ability in terms of interpolation. For this reason, a fair comparison indeed require for blind tests independent datasets that do not have any relation to the known datasets.

## Discussion

Rather than achieving a high level of accuracy on existing datasets, both an increase in knowledge and understanding of the driving forces and mechanisms for protein-nucleic acids binding are required in order to improve the accuracy on all datasets. Although most of the programs have been validated with curated datasets, we find that some of the programs do not consistently show high predictive ability on all the datasets. That is to say, these programs are poor at recapitulating the main factors key to protein-nucleic acid binding.

According to results on all the programs, some RNA binding site prediction programs (RBscore, RNAProSite, aaRNA and BindN+) show predictive ability in DNA binding site prediction, but hardly any DNA binding site prediction program demonstrates high level of accuracy in RNA binding site prediction. Thus, the key features of RNA binding residues and DNA binding residues are similar and can be better captured by RBP-based datasets than by DBP-based datasets. Besides, we find the most stable and accurate programs are aaRNA, RNAProSite and RBscore, all programs that take the advantage of protein structure, while other sequence-based programs are less stable in terms of both data set and distance cutoff, implying that important structural features cannot be fully captured by sequence-based programs making them less accurate.

The previous assessments of the predictive ability were mainly focused on two approaches, comparisons between previously reported results[4] and with small tests[6] (**Supplementary Note 8 in S1 Text**). However, as shown by this large-scale test, datasets can be biased and tests on one or a few test sets cannot testify the bottom line of predictive ability of a program. Also, comparisons with previously reported results are indirect, stressing the importance of large-scale tests and comparisons. This work has systematically benchmarked most currently existing programs in various aspects to complement the loophole of previous assessments. Further, we provide and regularly maintain all the test sets in this work on our web site allowing benchmarking of novel methods on all these data sets. Thus, new programs can be directly compared with all the existing programs and merits of the programs can be demonstrated in a straightforward manner.

Finally, we notice also that some existing binding site prediction approaches contain theoretical drawbacks: 1. For predictions leading to a binary classification, the mixing together of all proteins is arguable (**Supplementary Note 4 in S1 Text**); 2. The use of non-orthogonal but redundant features in prediction renders loose the relationships between feature and prediction (**Supplementary Note 10 in S1 Text**); 3. Slide-window approaches in sequence-based methods do not consider the real spatial environment of the residues (**Supplementary Note 9 in S1 Text**). Therefore, there is still room for the search for better alternatives.

In order to be useful to the research community, it is very important to make the prediction programs and web servers available and user-friendly. Some current programs require special computational skills, some are slow in efficiency and some require special formatted input, stressing the importance of ease-of-use and robustness of a prediction web server.

## Methods

### Datasets

All of the data sets were built based on protein-nucleic acid co-crystal structures extracted from the PDB. 23 RNA binding protein datasets and 16 DNA binding protein datasets were collected from previous studies and listed in **Table 3**. Some unreasonable cases were excluded from the assessment datasets: 1) the presence of a DBP in a RBP set (PDB ID 1a1v); 2) superseded PDB structures; 3) peptides shorter than 20 residues; 4) weak and uncertain nucleic acid binding proteins including those with less than three binding residues; 5) PDB chains containing only Cα atoms; 6) proteins constituted by two separate short peptides. Other two data sets of RBP and DBP after 2014 have been curated. Sequence similarity of 25% have been used as cutoff to remove redundancy from other data sets by PISCES[69]. These two data sets include 15RBP and 31 DBP respectively. All the data sets could be downloaded on website: http://ahsoka.u-strasbg.fr/nbench/.

**Table 3:** Summary of datasets used in tests.

Name	Protein#	After screening	Reference	Seq id	Str id	resolution
BindN_R107	107	95	[8]	25	NA	3.5
DR_bind1_R69	69	69	[42]	NA	CATH	3
DR_bind1_R81	81	79	[42]	NA	CATH	3
KYG_R86	86	85	[14]	50	NA	NA
PPRInt_R86	86	83	[23]	70	NA	3
PRNA_R205	205	189	[19]	25	NA	3
SRCPred_R160	160	124	[39]	25	NA	NA
PRBR_R180	180	142	[22]	25	NA	3.5
RNABindR_R106	106	100	[20,21]	NA	NA	NA
RNABindR_R109	109	100	[20,21]	30	NA	3.5
RNABindR_R144	144	137	[20,21]	NA	NA	NA
RNABindR_R147	147	138	[20,21]	30	NA	3.5
RNABindR_R198	198	187	[20,21]	30	NA	3.5
RNABindR_R111	111	101	[20,21]	30	NA	3.5
meta2_R44	44	44	[4]	40	NA	NA
aaRNA_R67	67	67	[27]	30	NA	NA
aaRNA_R141	141	136	[27]	25	NA	3
aaRNA_R205	205	200	[27]	25	NA	3
RBscore_R130	130	130	[28]	25	TMscore<0.7	3.5
RBscore_R116	117	116	[28]	25	TMscore<0.7	3.5
Sungwook_R267	267	178	[38]	60	NA	3
Sungwook_R727	727	574	[38]	NA	NA	3
Sungwook_R3149	3149	2632	[38]	NA	NA	3
New_R15	15	15		25	NA	5
BindN_D62	62	66	[8]	25	NA	NA
ProteDNA_D253	253	253	[70]	20	NA	3.5
Pro-dna_D99	99	188	[56]	20	NA	3
Hidetoshi_D52	52	49	[54]	NA	NA	3.2
Shandar_D140	140	138	[12]	25	NA	2.5
Susan_D56	56	54	[55]	NA	CATH	3
DBD-Hunter_D179	179	177	[59]	35	NA	3
Luscombe_D129	129	182	[71]	NA	NA	3
DBindR_D374	374	329	[51]	25	NA	3.5
DISPLAR_D428	428	390	[10]	50	NA	NA
DNABINDPROT_D54	54	50	[24]	NA	NA	NA
PreDNA_D224	224	216	[63]	25	NA	3
RBscore_D381	381	381	[28]	25	NA	3.5
metaDBSite_D232	232	225	[34]	30	NA	3
metaDBSite_D316	316	308	[34]	30	NA	3
SDCPred_D159	159	158	[52]	25	NA	2.5
New_D31	31	31		25	NA	5
RBscore_P627	628	627	[28]	NA	NA	3.5
All_P5114	5058	5058		NA	NA	NA

NA: not applicable

### Definition of binding sites

The minimum distance from any atom of a protein residue to any nucleic acid atom defines the distance from a protein residue and the nucleic acid. Nucleic acid binding sites are defined when such distances to the nucleic acid are shorter than certain thresholds. The range between 3.5 to 6Å, with 0.5Å step, was used as hierarchical thresholds to define binding residues in test sets. Besides, a nucleic acid binding residue always requires accessible surface area change (ΔASA>0Å^2^) upon complex formation with the nucleic acid. Accessible surface area is measured by NACCESS[72] with default parameters.

### Assessment criteria

Receiver Operating Characteristic (ROC) curve together with Area Under Curve (AUC) is always used as criterion for accuracy[73]. We define the accuracy of a set of proteins by averaging accuracies of all proteins. We suggest the weighted arithmetic mean of AUC (wAUC) and mean of AUC (mAUC) as two criteria of accuracy for a set of proteins:

$$\text{wAUC}=\frac{\sum \;AUC\left(i\right)\times len\left(i\right)}{\sum \;len\left(i\right)}$$ (1)

$$\text{mAUC}=\bar{AUC}=\frac{\sum \;AUC\left(i\right)}{N}$$ (2)

For a protein i, AUC(i) is its AUC value and len(i) is length of the protein, while N is the number of proteins in a dataset. We call the AUC that compare all the residues in a dataset together as total AUC (tAUC) and use it as a reference for comparison.

We define standard deviation of AUC of a data set as sAUC to show the accuracy stability varying the data sets:

$$\text{sAUC}=\sqrt{\frac{\sum \;AUC\left(i\right)\times AUC\left(i\right)-N\times {\bar{AUC}}^{2}}{N-1}}$$ (3)

Other binary criteria include specificity, sensitivity, precision, accuracy, F1 score and Matthews correlation coefficient:

$$\text{specificity}=\frac{\text{TP}}{\text{TP}+\text{FN}}$$ (4)

$$\text{sensitivity}=\frac{\text{TN}}{\text{FP}+\text{TN}}$$ (5)

$$\text{precision}=\frac{\text{TP}}{\text{TP}+\text{FP}}$$ (6)

$$\text{accuracy}=\frac{\text{TP}+\text{TN}}{\text{P}+\text{N}}$$ (7)

$$\text{F}1=\frac{2\text{TP}}{2\text{TP}+\text{FP}+\text{FN}}$$ (8)

$$\text{MCC}=\frac{\text{TP}\times \text{TN}+\text{FP}\times \text{FN}}{\sqrt{\left(TP+FP)(TP+FN)(TN+FP)(TN+FN\right)}}$$ (9)

TP is true positive, TN is true negative, FP is false positive and FN is false negative. P is total positive and N is total negative.

MAVR, maximum accuracy variation rate, is defined as:

$$\text{MAVR}=\text{max}(\frac{\Delta wAUC}{\Delta d})$$ (10)

Δd is the difference between two distance cutoffs used to define binding sites. ΔwAUC is the resulted accuracy variance defined by wAUC, this can also be replaced by mAUC or tAUC.

MAV=max(ΔwAUC) (11)

MAV is the maximum accuracy variation and CAVR is the cumulated accuracy variation rate:

$$\text{CAVR}=\sum_{d=3.5Å}^{6.0Å}\frac{\Delta wAUC}{\Delta d}$$ (12)

MAVR, MAV and CAVR are criteria to assess the distance cutoff dependent variation in accuracy.

The correlation coefficient between prediction score and minimum distance to nucleic acid is defined by the Pearson correlation coefficient:

$$\rho =\frac{cov(\text{min}(d),s)}{\sigma \text{min}(d)\sigma s}$$ (13)

min(d) is the minimum distance from a residue to any nucleic acid atom, s is the prediction score, cov is the covariation and σx is the standard deviation.

### Use of prediction programs

Li lab and Xiaoyong Pan provided RNAProSite and xypan prediction results respectively, with default parameter. aaRNA was used by inputting the structure file with default parameters. RNABindR and RNABindRPlus were predicted with default parameters, while removing 95% sequence identity in RNABindRPlus. KYG was predicted with command line using “method_type = 8” option. RBscore_SVM was based on the training set of R246. DBS-PSSM, DBS-Pred, RBRIdent, PPRInt, PRBR, DNABind, RBRDetector and ProteDNA were used with default parameter. The DISPLAR program was provided by Sanbo Qin and was run with default parameters. BindN and BindN+ were used by default parameters, while suffix “_RNA” and “_DNA” are RNA mode and DNA mode respectively. PRNA and Predict_RBP were trained on PRNA_R205 and BindN_R107 respectively, without cross-validation and applied with default parameters. DNABINDPROT was predicted with option “Fast 1” and DR_bind1 was predicted in the RNA mode. N-terminal and C-terminal residues not predicted by PRBR are taken as non-binding sites and assigned 0 as prediction score. For binary predictions of DNABINDPROT, DISPLAR, DBS-Pred and DBS-PSSM, positive sites are assigned a prediction score of 1, while negatives are assigned 0.

### Availability

All data in this assessment are available on NBench web site http://ahsoka.u-strasbg.fr/nbench/.

## Supporting Information

S1 Text Supplementary Note 1–12. (DOCX)

S1 Table Percentage of binding sites defined by different distance cutoffs on all data sets. (XLSX)

S2 Table Accuracy variation resulted from definition of binding sites. Binding sites are defined by 3.5Å and 6Å defined sites are taken as prediction, accuracies are assessed and vice versa.
(XLSX)

S3 Table Results of DR_bind1 and RBRDetector. (XLSX)

S1 Fig Mean AUC performance of all programs on all data sets. (EPS)

S2 Fig Total AUC performance of all programs on all data sets. (EPS)

S3 Fig MAVR, MAV and CAVR based on mean AUC. (EPS)

S4 Fig MAVR, MAV and CAVR based on total AUC. (EPS)

S5 Fig Specificities of all programs on all data sets. (EPS)

S6 Fig Sensitivities of all programs on all data sets. (EPS)

S7 Fig Precisions of all programs on all data sets. (EPS)

S8 Fig Accuracies of all programs on all data sets. (EPS)

S9 Fig F1 scores of all programs on all data sets. (EPS)

S10 Fig Matthews correlation coefficients of all programs on all data sets. (EPS)

S11 Fig A) RBscore_SVM approach performance trained on 130 RBP with cross-validation. B) RBscore_SVM approach performance trained on 246 RBP data set with cross-validation. C) RBscore_SVM approach performance trained on 381 DBP data set with cross-validation. D) RBscore_SVM approach performance trained on 627 NBP data set with cross-validation. E) Comparison of RBscore_SVM approach performance trained on different data sets. F) Comparison of RBscore_SVM approach performance with top rank prediction programs. (EPS)

S12 Fig Percentage of nucleic acid binding sites defined by different distance cutoffs on RBscore_P627 data sets. Distributions on other data sets are available as zip file on NBench website.
(EPS)

S13 Fig Scheme to illustrate that a slide-window approach cannot consider fully a real neighboring environment. Case of poly(A)-binding protein (PDB id: 1cvj chain A). Residue F102 is mutated into His, Glu and Asp. Variations around F102 is easy to comprehend but two other regions close in space are also related. RBscore consider the spatial neighbors, and show difference in region 127–129 and region 172–179. BindN+ only show certain difference in the region 172–179. PPRInt and RNABindRPlus hardly show difference in prediction. As demonstrated by deep mutational scanning, the single point mutation to Glu and Asp obviously make difference in the binding and should also affect other residue neighbors. With a sequence based slide-window approach it is difficult to capture such structural differences.
(EPS)

S14 Fig Scheme to illustrate the redundancy of ‘New feature’ by just mapping to feature values. Approach 1 is to input directly the residue information into the learning machine, approach 2 is first to map the residue information to a function and input the resulted feature vector into the learning machine. If we take the mapping step together with the learning machine as another learning machine, approach 2 is not different from approach 1.
(EPS)

S15 Fig Variations of accuracy with the distance cutoff used to define the binding sites. The plot is based on test on data set ‘DR_bind1_R69’. X-axis show the distance cutoff used to define nucleic acid binding sites, while Y-axis show the resulted wAUC value. We find the wAUC values vary for different distance cutoffs, and the distributions for different programs are different.
(EPS)
