# Supplementary material for: A Large-Scale Assessment of Nucleic Acids Binding Site Prediction Programs
Source: PLoS Comput Biol. 2015 Dec 17;11(12):e1004639. doi: 10.1371/journal.pcbi.1004639 (PMC4683125; doi:10.1371/journal.pcbi.1004639)
Supplement: S1 Text — (DOCX) [file pcbi.1004639.s001.docx]

**A large-scale** **assessment of nucleic acids binding site prediction programs**

Zhichao Miao and Eric Westhof*

Architecture et Réactivité de l'ARN, Université de Strasbourg, Institut de biologie moléculaire et cellulaire du CNRS, 15 Rue Descartes 67000 Strasbourg France

* Corresponding author. E-mail: [e.westhof@ibmc-cnrs.unistra.fr](mailto:e.westhof@ibmc-cnrs.unistra.fr)

**Abstract**

Computational prediction of nucleic acid binding sites in proteins are necessary to disentangle functional mechanisms in most biological processes and to explore the binding mechanisms. Several strategies have been proposed, but the state-of-the-art approaches display a great diversity in i) the definition of nucleic acid binding sites; ii) the training and test datasets; iii) the algorithmic methods for the prediction strategies; iv) the performance measures and v) the distribution and availability of the prediction programs. Here we report a large-scale assessment of 19 web servers and 3 stand-alone programs on 41 datasets including more than 5000 proteins derived from 3D structures of protein-nucleic acid complexes. Well-defined binary assessment criteria (specificity, sensitivity, precision, accuracy…) are applied. We found that i) the tools have been greatly improved over the years; ii) some of the approaches suffer from theoretical defects and there is still room for sorting out the essential mechanisms of binding; iii) RNA binding and DNA binding appear to follow similar driving forces and iv) dataset bias may exist in some methods.

**Supplementary Note 1**. Gap between sequence number and functional site annotation.

Currently, the Protein databank includes more than 5000 DNA/RNA-protein complex structures that is ~800 non-redundant DNA/RNA binding protein chains (below 25% sequence identity)[[1](#_ENREF_1)]. Compared with the number of protein structures in the database, the protein-nucleic acid (NA) complexes take up only 5%. Even if the protein structures form >90% of the structure database[[2](#_ENREF_2)], only less than 1/1000th proteins of known sequences have experimental structures available[[3](#_ENREF_3)]. Given the vast number of DNA/RNA-protein interactions in bio-systems, it is likely that only a very small part of such interactions have been characterized. Therefore, computational prediction methods are of great importance, but the limited structural data also implies that predictions that do not consider fundamental rules of protein-nucleic acid binding would result in database bias.

**Supplementary Note 2**. DNA binding and RNA binding.

DNA is normally double-stranded and in DNA-protein complexes, the protein binding interface generally lie on the grooves formed by the backbones of nucleic acids with specific interactions to the Watson-Crick pairs. On the opposite, more single-stranded structures are found in RNA-protein complexes. In several RNA structures, both double- and single-stranded parts are present with some of the single-stranded segments organized as RNA modules where Watson-Crick, non-Watson-Crick and stacking stabilize the structure and contribute to specific protein binding. Although differences between protein binding interfaces on DNA and RNA can be clearly illustrated, difference between DNA and RNA binding interfaces on protein and the difference between the driving forces of DNA- and RNA- binding are yet to be determined. Currently, RNA- and DNA-binding residue predictions are always treated as different problems, or trained with different datasets within the same model[[4-7](#_ENREF_4)]. But, when we applied the same algorithm on both DNA and RNA binding proteins (DBP and RBP), we observed that most of the programs exhibit certain prediction abilities on both types on proteins, some with AUC values >0.7 on all the datasets. Thus, in both DBP and RBP similar interaction rules during nucleic acid binding are operating. It is therefore necessary to test and compare the programs on both DBP and RBP datasets together to provide more information and to extend our comprehension.

**Supplementary Note 3**. Definition of nucleic acid binding sites based on a PDB structural complex.

To define nucleic acid binding sites based on a PDB complex structure, a first approach is the use of distance cutoff, which is easy to implement and is adopted by a large part of the studies. The distance cutoff approach is such that when the minimum distance between any atom in a protein residue and any nucleic acid atom is below a certain fixed value, the residue is defined as a binding site. The method is simple, but it may retrieve some totally buried residues as binding sites, especially when the distance cutoff is chosen too long. The currently used cutoffs vary between 3.5[[5](#_ENREF_5),[6](#_ENREF_6),[8](#_ENREF_8),[9](#_ENREF_9)] , 5, 6 to 7Å[[10](#_ENREF_10),[11](#_ENREF_11)], which may lead to very different definitions. The second approach is to use a structure analysis program like ENTANGLE[[12-15](#_ENREF_12)] to define different types of interactions including hydrogen bonding and Van de Waals interaction. Similar programs such as HBPLUS[[16](#_ENREF_16)] and NUCPLOT[[17](#_ENREF_17)] are also used by some of the groups[[18](#_ENREF_18),[19](#_ENREF_19)], but still cannot constitute a general criteria. Other methods consider accessible surface area change (ΔASA) to define nucleic acid binding sites. The cutoff can be either 0, 0.1Å^2^ or 10% area change.

Normally, a distance within 3.5Å should be reasonable for specific interactions that include hydrogen bonds or salt bridges. But water-mediated hydrogen bonds present longer distances around ~4.0Å, and some van der Waals contacts may also have longer interaction distances. Although specific interactions within short distance is more important, it is not sure if all of these interactions are necessary to take effect at the same time in binding. Distances longer than 6Å definitely include some non-specific interactions. But such non-specific interactions may be useful during the binding process and may help the formation of a funnel to guide the ligand sliding into the binding center. The residues around the binding interface should not change abruptly from binding sites to non-binding sites.

Furthermore, binary definitions based on a single PDB structure reflect a single state of the protein and do not constitute, per se, a gold standard for assessment. Well-known cases include the moonlighting proteins where the multiple functions can originate from different binding sites. Another case is a protein with a single point mutation that maintains the same structure but disables binding.

**Supplementary Note 4**. Should one compare all proteins together?

Current prediction methods for nucleic acid binding residues normally compare all residues in all proteins together to measure the area under the ROC curve (AUC) and other binary metrics (sensitivity, specificity, accuracy et al.) for assessment, which is a by-product of machine learning approaches[[20](#_ENREF_20)]. However, different nucleic acid binding proteins may have different affinities to nucleic acids [[21](#_ENREF_21)] and, thus, it is still arguable if there is a universal cutoff for all residues from all proteins. For binding sites prediction, the main aim is to locate the key binding region rather than all the details of binding sites, and the only necessity is to discriminate binding residues against non-binding residues in the same protein. Thus, the current measurement, which uses total AUC to compare all residues from all proteins together, may bias the accuracy by overemphasizing comparisons between residues of different proteins. An alternative is to measure AUCs for each protein and average over a data set as the final accuracy. We suggest weighted averaged AUC and averaged AUC values (wAUC and mAUC) as assessment criteria, with the total AUC (tAUC) as a reference. Further, binary assessment criteria normally require a fixed cutoff value, which is a trade-off between specificity and sensitivity, to determine whether a residue is predicted as binding site or not. In this way, we can find a program with similar wAUC/mAUC on two datasets, but its sensitivity on one data set is much higher than the other, while the other data set is higher in specificity. For example, RBscore and aaRNA on meta_R44 and Sungwook_R267. Thus, binary accuracies also change with the cutoff value.

**Supplementary Note 5**. *The two main approaches in nucleic acid binding site prediction.*

*1) Machine Learning based approaches*

Owing to the strong capacity in dealing with big data, machine learning is a widely used approach in functional site prediction, not only for DNA-/RNA-binding sites but also for transcription factor (TF) binding sites[[22](#_ENREF_22)], ligand binding sites[[23](#_ENREF_23)], etc. Machine learning automatically generalize rules from training data to make prediction models. The machine learning techniques used in previous predictions include SVM[[5](#_ENREF_5),[6](#_ENREF_6),[11](#_ENREF_11),[12](#_ENREF_12),[24-30](#_ENREF_24)], decision tree[[4](#_ENREF_4)], Naïve Bayes[[31-33](#_ENREF_31)], artificial neuron network[[7-9](#_ENREF_7),[34](#_ENREF_34),[35](#_ENREF_35)] and random forest[[15](#_ENREF_15),[36](#_ENREF_36),[37](#_ENREF_37)]. Since the learning technique is also a very important aspect in binding site prediction, programs of different learning techniques need to be comprehensively compared.

Normally, machine learning is very powerful in terms of interpolation but cannot promise a prediction of extrapolation. Because the training sets are finite and the unknown data is uncertain, learning theory normally does not yield guarantees of prediction performance unless the training data includes most of the folds. The known complex structures are still very few compared with known sequence space (**Supplementary Note 1**), while the set of solved structures is not random sampling since it is strongly dependent on the ease of crystallization. Perspective progress could be made through either validating the completeness of training data in data space and train unbiased models or seeking alternative approaches that could describe the essence of protein-nucleic acid binding.

*2) Template-based approaches*

The template-based approach[[11](#_ENREF_11),[38](#_ENREF_38),[39](#_ENREF_39)], which can be traced back to the threading approach in protein structure prediction and structure alignment, was also applied. While a learner of machine learning attempts to generalize common rules from known experience of a dataset, a template-based approaches tries to extract specific rules from single cases. They may combine binding site prediction with protein type (RBP/DBP) prediction, since they predict complex structures between protein and nucleic acids that can be used to annotate binding sites[[1](#_ENREF_1)]. The accuracy of these programs strongly depends on protein structure alignment program.

Another idea from template-based approaches is that the biologist would like to have the most accurate binding sites data when binding sites are already known in homologous structures[[24](#_ENREF_24)]. Then, these methods can directly adopt the known knowledge without generalization and also achieve the highest accuracies. Recent RNABindRPlus[[24](#_ENREF_24)], DNABind[[40](#_ENREF_40)], RBRDetector[[41](#_ENREF_41)] and ProteDNA[[42](#_ENREF_42)] work integrated both template-based approach and machine learning approach together to obtain better prediction.

**Supplementary Note 6**. *Accuracy variation attributed to distance cutoff.*

Nucleic acid binding sites are defined by hierarchical distance cutoffs for the analysis. As shown in **Table S1** and **S12 Figure**, the number of binding sites defined by a distance cutoff of 6Å is almost two times that defined by 3.5Å. And the requirement of ΔASA>0Å^2^ obviously excludes some buried and non-binding residues. Distance cutoffs longer than 6Å would certainly include buried residues (even all the residues in a protein may be then defined as binding sites), since the binding site number is reduced by ~5% under the ΔASA>0Å^2^ requirement. Distributions of number of binding sites on other 41 datasets differ in the numbers (all distribution plots are available as **Supplementary**) but the conclusions remain the same.

To understand the differences in accuracy resulting from the value of the distance cutoff, we use the binding sites defined by 3.5Å and 6Å as two predictions and tested with hierarchical distance cutoffs as gold standards (binding sites defined by hierarchical cutoffs). ΔASA>0Å^2^ is required for all the binding site, and the prediction values are given as 1 for binding sites and 0 for non-binding sites. In **Table S2**, we can find when 3.5Å is used for prediction and 6Å for defining the binding sites, the resulted specificity is 100% but the sensitivity is low as 51-62% (high false negative rate) with the total accuracy (ACC) ~90% (87% as minimum). Reversely, when 6Å is used for prediction and 3.5Å for defining the binding sites, sensitivity is 100% while specificity and precision drop to ~90% and 52-65% respectively, giving the same total accuracy. This demonstrates that distance cutoff variation could result in an accuracy shift as high as 10%, and such a gap may become even larger without considering the requirement of ΔASA>0Å^2^. Thus, a program may exhibit low accuracy performance just because a different distance cutoff used. This difference also exists for other criteria such as AUC (area under ROC curve), precision and F1 score. To get a more complete view of prediction performance, it could be helpful to show the accuracy distribution as a landscape with the distance cutoff as a variable parameter.

To avoid unreasonable definitions, such as buried residues defined as binding sites, we propose ΔASA>0Å^2^ as a prerequisite for nucleic acid binding sites and use distance cutoffs from 3.5 to 6Å with a step of 0.5Å to make hierarchical definitions for the assessments so that all the programs could be compared on the same basis.

**Supplementary Note 7**. *Size of training set, stability of prediction accuracy and independent test.*

As shown in **S11 Figure**, we trained simple SVM based prediction models based on different data sets using 5-fold cross-validation. We find the cross-validation models show identical accuracy distributions on all the data sets no matter what training set used. And training with the whole data set is similar to the training with 4 fold data out of 5. And we compare the trainings of different data sets in **Fig S11E**, we can find no training can show stable performance on all the data sets. This illustrates that the size of the training set can result in accuracy fluctuations but larger data set cannot result in stable but high predictions on all data sets. None of the SVM models shows a prediction as stable as RBscore, RNAProSite or aaRNA.

Independent tests show best the predictive ability of a program. As nearly all programs use pdb data that appeared before 2014, datasets after 2014 are ‘blind’ tests for all the programs. Over-trained programs cannot show high prediction on independent datasets. And we find the performances on new data sets after 2014 (New_R15 and New_D31, which are not included in the training sets) are always low, ~0.7 wAUC. Although training with larger dataset (RBscore_P628 model) can show slightly higher in accuracy, the accuracy cannot reach the accuracy level of other data sets. This also implies that the new datasets can be used for assessment. As compared with RBscore, RNAProSite and aaRNA, which are more stable in accuracy, the simple SVM based prediction models and other methods that have similar distribution are more biased.

**Supplementary Note 8**. *Cross-validation with small-scale test.*

Many previously reported prediction works pointed out useful features by using cross-validation or some small-scale independent tests. However, as shown by recent work^[^[^43^](#_ENREF_43)^]^, two assumptions need to be fulfilled before cross-validation: 1) the number of folds should not be too small; 2) the data should be the result of random sampling. Besides, sequence identity alone is not enough to remove structural similarity. For example, we find 50s ribosomal protein L25 from PDB 1dfu chain P and 1feu chain A are identical RMSD 1.37Å but only share 16.9% sequence identity. Small-scale independent testset without considering structural similarity is hardly reliable.

Comparing two improved versions of PRNA[[44](#_ENREF_44)], xypan[[45](#_ENREF_45)] and RBRIdent[[46](#_ENREF_46)] both of which use cross-validation and small-scale tests, with PRNA on all the datasets, the improvement of xypan over PRNA is not obvious while RBRIdent is much lower in accuracy. Therefore, it could be dangerous to make judgement on the effectiveness of a prediction with simply cross-validation and small-scale tests.

**Supplementary Note 9**. *Drawbacks of the slide-window approach and template based approach.*

Many sequence-based predictions use a slide-window approach, which is easy to implement. The essence of slide-window approach can be traced back to the fragment-based prediction of protein structure, which underlies the homologous search of fragment. However, the problem of binding site prediction is different from protein structure prediction. And residues close in space that form the binding interface can be far away in sequence[[47](#_ENREF_47)] (**S13 Figure**). In a slide-window scheme, residue neighbors far away in sequence cannot be considered. But the neighboring residues form the environment of the target residue and can determine the binding. Slide-window approach may result in a dangerous situation that the prediction of a target residue can stay the same even all its spatial neighbors have been mutated. This also explains the reason why structure-based predictions are more accurate than sequence based ones. And sequence-based predictions still have room for improvement to avoid slide window approach and fill this gap.

Similarly, a template-based approach is also based on homologous search and may directly use the binding sites of the template. However, when some of the neighbor residues have been mutated, to a different binding results, which cannot be captured by this approach.

**Supplementary Note 10**. *Repeated features that are non-orthogonal and parameter number of prediction model.*

With the frequent use of machine learning in binding site prediction, many different features have been proposed. But the relationship between the features and the prediction is loose, because of the complicated learning process. Further, many of the features, who are derived in similar ways are not orthogonal with each other and cannot stand for different aspects of protein-nucleic acid binding. The metric structure of the data space is altered by non-orthogonal features and it, thus, will contain redundant information[[48](#_ENREF_48)]. Therefore, some of the features are not effective enough. For instance, some prediction approaches map the sequence to AA index values. Then, a residue is expressed as a feature vector of values. This is an easy process to implement in machine learning. But 20 amino acid types only result in 20 different vectors to the learning machine. If a learning machine can always reach the most optimized model, such mapping process makes no different from directly taking the residue type as input (**S14 Figure**). The learning process together with the mapping process can be taken as a big learning machine. If such a method is assessed by cross-validation with small scale independent test (**Supplementary Note 8**), conclusion could be misleading. Therefore, it is better to use orthogonal features derived in different ways. For example, electrostatics potential could be derived from structure-based calculation, evolutionary information derived from homologous sequence search and alignment.

Furthermore, complicated prediction models with many parameters could capture more specific rules of the training set than capture the essential general rules. These models are only able to show interpolation ability and be less predictive.

**Supplementary Note 11**. *Programs favor the distance cutoff used for their training.*

Distance cutoff difference certainly leads to different nucleic acid binding sites (**Supplementary Note 6**) and the prediction accuracies are also influenced (**Fig 1**). How the wAUC changes with distance cutoff is shown in **S15 Figure**, which is a test on dataset DR_bind1_R69. As BindN+[[6](#_ENREF_6)], RBscore and aaRNA[[35](#_ENREF_35)] use 3.5Å as distance cutoff, their accuracies drop from 3.5 to 6Å, while for RNABindRPlus[[24](#_ENREF_24)] who uses 5Å as distance cutoff, its accuracy increases from 3.5 to 5Å and drops thereafter. PPRInt[[26](#_ENREF_26)] uses 6Å as distance cutoff, and its accuracy slightly increases from 3.5 to 6Å. Generally, a program favors the distance cutoff used in training. Distributions on all other datasets differ in the numbers but the conclusions stay the same.

**Supplementary Note 12**. *Use of the programs.*

Predict_RBP and PRNA require special computational skills and a re-training of the prediction model before using the programs, making it very difficult to use even for computationally trained researchers. Thus, webserver is a better choice for distribution. Besides, efficiency is very important for a prediction web server. DR_bind1[[18](#_ENREF_18)] and RBRDetector[[41](#_ENREF_41)] are slow and cannot handle prediction of all the data set. So it is not easy to describe their predictive abilities. Still, some existing programs have special requirements for the input and cannot deal with all types of predictions. RNAProSite can only predict 4900 protein chains out of the 5114 cases, DNABINDPROT does not treat with chains in lower cases. Some programs do not recognize selenomethionine which is a common replacement of methionine in crystallography used to determine phase, leading to shorter and fragmented prediction which is unreasonable. Therefore, more efforts should be emphasized in developing efficient and robust web servers.

**References**

1. Zhao HY, Yang YD, Zhou YQ (2013) Prediction of RNA binding proteins comes of age from low resolution to high resolution. Mol Biosyst 9: 2417-2425.

2. Berman HM, Westbrook J, Feng Z, Gilliland G, Bhat TN, et al. (2000) The Protein Data Bank. Nucleic Acids Res 28: 235-242.

3. Moult J (2008) Comparative modeling in structural genomics. Structure 16: 14-16.

4. Carson MB, Langlois R, Lu H (2010) NAPS: a residue-level nucleic acid-binding prediction server. Nucleic Acids Research 38: W431-W435.

5. Wang LJ, Brown SJ (2006) BindN: a web-based tool for efficient prediction of DNA and RNA binding sites in amino acid sequences. Nucleic Acids Research 34: W243-W248.

6. Wang LJ, Huang CY, Yang MQ, Yang JY (2010) BindN plus for accurate prediction of DNA and RNA-binding residues from protein sequence features. Bmc Syst Biol 4.

7. Tjong H, Zhou HX (2007) DISPLAR: an accurate method for predicting DNA-binding sites on protein surfaces. Nucleic Acids Research 35: 1465-1477.

8. Ahmad S, Gromiha MM, Sarai A (2004) Analysis and prediction of DNA-binding proteins and their binding residues based on composition, sequence and structural information. Bioinformatics 20: 477-486.

9. Ahmad S, Sarai A (2005) PSSM-based prediction of DNA binding sites in proteins. Bmc Bioinformatics 6.

10. Kim OTP, Yura K, Go N (2006) Amino acid residue doublet propensity in the protein-RNA interface and its application to RNA interface prediction. Nucleic Acids Research 34: 6450-6460.

11. Shulman-Peleg A, Shatsky M, Nussinov R, Wolfson HJ (2008) Prediction of interacting single-stranded RNA bases by protein-binding patterns. J Mol Biol 379: 299-316.

12. Wang Y, Xue Z, Shen G, Xu J (2008) PRINTR: Prediction of RNA binding sites in proteins using SVM and profiles. Amino Acids 35: 295-302.

13. Wang CC, Fang YP, Xiao JM, Li ML (2011) Identification of RNA-binding sites in proteins by integrating various sequence information. Amino Acids 40: 239-248.

14. Allers J, Shamoo Y (2001) Structure-based analysis of Protein-RNA interactions using the program ENTANGLE. J Mol Biol 311: 75-86.

15. Freddolino PL, Harrison CB, Liu Y, Schulten K (2010) Nat Phys 6: 751.

16. Mcdonald IK, Thornton JM (1994) Satisfying Hydrogen-Bonding Potential In Proteins. J Mol Biol 238: 777-793.

17. Luscombe NM, Laskowski RA, Thornton JM (1997) NUCPLOT: a program to generate schematic diagrams of protein-nucleic acid interactions. Nucleic Acids Res 25: 4940-4945.

18. Chen YC, Wu CY, Lim C (2007) Predicting DNA-binding amino acid residues from electrostatic stabilization upon mutation to Asp/Glu and evolutionary conservation. Proteins 67: 671-680.

19. Ozbek P, Soner S, Erman B, Haliloglu T (2010) DNABINDPROT: fluctuation-based predictor of DNA-binding residues within a network of interacting residues. Nucleic Acids Res 38: W417-W423.

20. Joachims T (1999) Making large-scale support vector machine learning practical. Advances in kernel methods: MIT Press. pp. 169-184.

21. Yang XF, Li HT, Huang YY, Liu SY (2013) The dataset for protein-RNA binding affinity. Protein Sci 22: 1808-1811.

22. Maienschein-Cline M, Dinner AR, Hlavacek WS, Mu FP (2012) Improved predictions of transcription factor binding sites using physicochemical features of DNA. Nucleic Acids Res 40.

23. Wong GY, Leung FHF, Ling SH (2013) Predicting Protein-Ligand Binding Site Using Support Vector Machine with Protein Properties. Ieee Acm T Comput Bi 10: 1517-1529.

24. Walia RR, Xue LC, Wilkins K, El-Manzalawy Y, Dobbs D, et al. (2014) RNABindRPlus: A Predictor that Combines Machine Learning and Sequence Homology-Based Methods to Improve the Reliability of Predicted RNA-Binding Residues in Proteins. Plos One 9.

25. Bhardwaj N, Lu H (2007) Residue-level prediction of DNA-binding sites and its application on DNA-binding protein predictions. Febs Lett 581: 1058-1066.

26. Kumar M, Gromiha AM, Raghava GPS (2008) Prediction of RNA binding sites in a protein using SVM and PSSM profile. Proteins 71: 189-194.

27. Li T, Li QZ, Liu S, Fan GL, Zuo YC, et al. (2013) PreDNA: accurate prediction of DNA-binding sites in proteins by integrating sequence and geometric structure information. Bioinformatics 29: 678-685.

28. Wang DD, Li TH, Sun JM, Li DP, Xiong WW, et al. (2013) Shape string: A new feature for prediction of DNA-binding residues. Biochimie 95: 354-358.

29. Li BQ, Feng KY, Ding J, Cai YD (2014) Predicting DNA-binding sites of proteins based on sequential and 3D structural information. Mol Genet Genomics 289: 489-499.

30. Park B, Im J, Tuvshinjargal N, Lee W, Han K (2014) Sequence-based prediction of protein-binding sites in DNA: Comparative study of two SVM models. Comput Meth Prog Bio 117: 158-167.

31. Terribilini M, Sander JD, Lee JH, Zaback P, Jernigan RL, et al. (2007) RNABindR: a server for analyzing and predicting RNA-binding sites in proteins. Nucleic Acids Research 35: W578-W584.

32. Towfic F, Caragea C, Gemperline DC, Dobbs D, Honavar V (2010) Struct-NB: predicting protein-RNA binding sites using structural features. Int J Data Min Bioin 4: 21-43.

33. Yan CH, Terribilini M, Wu FH, Jernigan RL, Dobbs D, et al. (2006) Predicting DNA-binding sites of proteins from amino acid sequence. Bmc Bioinformatics 7.

34. Fernandez M, Kumagai Y, Standley DM, Sarai A, Mizuguchi K, et al. (2011) Prediction of dinucleotide-specific RNA-binding sites in proteins. Bmc Bioinformatics 12.

35. Li S, Yamashita K, Amada KM, Standley DM (2014) Quantifying sequence and structural features of protein–RNA interactions. Nucleic Acids Res 10.1093/nar/gku681.

36. Wu JS, Liu HD, Duan XY, Ding Y, Wu HT, et al. (2009) Prediction of DNA-binding residues in proteins from amino acid sequences using a random forest model with a hybrid feature. Bioinformatics 25: 30-35.

37. Ma X, Guo J, Wu JS, Liu HD, Yu JF, et al. (2011) Prediction of RNA-binding residues in proteins from primary sequence using an enriched random forest model with a novel hybrid feature. Proteins 79: 1230-1239.

38. Gao M, Skolnick J (2008) DBD-Hunter: a knowledge-based method for the prediction of DNA-protein interactions. Nucleic Acids Research 36: 3978-3992.

39. Zhao HY, Yang YD, Zhou YQ (2011) Structure-based prediction of RNA-binding domains and RNA-binding sites and application to structural genomics targets. Nucleic Acids Research 39: 3017-3025.

40. Liu R, Hu J (2013) DNABind: a hybrid algorithm for structure-based prediction of DNA-binding residues by combining machine learning- and template-based approaches. Proteins 81: 1885-1899.

41. Yang XX, Deng ZL, Liu R (2014) RBRDetector: improved prediction of binding residues on RNA-binding protein structures using complementary feature- and template-based strategies. Proteins 82: 2455-2471.

42. Chu WY, Huang YF, Huang CC, Cheng YS, Huang CK, et al. (2009) ProteDNA: a sequence-based predictor of sequence-specific DNA-binding residues in transcription factors. Nucleic Acids Res 37: W396-W401.

43. Braga-Neto UM, Zollanvari A, Dougherty ER (2014) Cross-validation under separate sampling: strong bias and how to correct it. Bioinformatics 10.1093/bioinformatics/btu527.

44. Liu ZP, Wu LY, Wang Y, Zhang XS, Chen LN (2010) Prediction of protein-RNA binding sites by a random forest method with combined features. Bioinformatics 26: 1616-1622.

45. Pan X, Zhu L, Fan Y-X, Yan J (2014) Predicting protein–RNA interaction amino acids using random forest based on submodularity subset selection. Computational Biology and Chemistry 53, Part B: 324-330.

46. Xiong D, Zeng J, Gong H (2015) RBRIdent: An algorithm for improved identification of RNA-binding residues in proteins from primary sequences. Proteins 10.1002/prot.24806.

47. Melamed D, Young DL, Gamble CE, Miller CR, Fields S (2013) Deep mutational scanning of an RRM domain of the Saccharomyces cerevisiae poly(A)-binding protein. Rna 19: 1537-1551.

48. Xu Y, Furao S, Zhao J, Hasegawa O (2009) To obtain orthogonal feature extraction using training data selection. Proceedings of the 18th ACM conference on Information and knowledge management. Hong Kong, China: ACM. pp. 1819-1822.
